# Ten quick tips for protecting health data using de-identification and perturbation of structured datasets

**DOI:** 10.1371/journal.pcbi.1013507

**Published:** 2025-09-23

**Authors:** Tshikala Eddie Lulamba, Themba Mutemaringa, Nicki Tiffin

**Affiliations:** 1 South African National Bioinformatics Institute, University of the Western Cape, Bellville, South Africa; 2 Provincial Health Data Centre (PHDC), Western Cape Department of Health and Wellness, Cape Town, South Africa; 3 Computational Biology Division, Integrative Biomedical Sciences Department, University of Cape Town, Cape Town, South Africa; SIB Swiss Institute of Bioinformatics, SWITZERLAND

## Abstract

Structured patient data generated within the health data ecosystem are shared both internally for operational use and also externally for research and public health benefit. Protecting individual privacy and health data confidentiality in these contexts relies on data de-identification and anonymisation, although there are no universally accepted standards for these processes and the techniques involved can be technically complex. We present practical recommendations grounded in the principle of data minimisation—avoiding unnecessary granularity and identifying variables that could lead to re-identification when combined with other datasets. We provide practical guidance for anonymising and perturbing structured health data in ways that support compliance with data protection laws, describing technical and operational methods for reducing re-identification risk that include rounding numerical values, replacing precise values with ranges, adding jitter to numeric fields, aggregating data, management of date values and separating sensitive fields from identifying data to prevent linkage leading to re-identification. While some methods require advanced technical knowledge, we focus here on accessible strategies that can be implemented without specialist expertise, recognising the importance of the legal and governance frameworks in which anonymisation occurs. These guidelines support researchers, data managers and institutions in sharing health data responsibly, maintaining data utility while upholding privacy and promoting ethical and legal data stewardship for data-driven health research.

## Author summary

Healthcare systems and health research programmes collect large amounts of patient data that are often shared both within organisations and across institutional boundaries. Health data are highly sensitive, and it is essential to ensure that individuals cannot be identified or recognised through the use of their health information. Data de-identification and anonymisation are the most common approaches for protecting individuals’ privacy and confidentiality in these settings, but there are no universal standards for these processes and they can be technically complex to apply. Here we describe practical, accessible technical and operational security measures that can be used to de-identify and anonymise structured health data in ways that comply with data protection laws. These practical guidelines can support data analysts and researchers working with sensitive health data, including those without prior experience in data anonymisation, to implement effective privacy-preserving techniques, including perturbation, for large, structured health-related datasets.

## Introduction

Healthcare systems collect extensive and varied personal medical data to document and manage client health over time and to provide continuity of care. The collected data include health histories and clinical details that can also be repurposed for public health surveillance, evidence-based policy-making and epidemiological research, providing insights into aggregated health trends and outcomes. These data may therefore be shared internally for operational review, as well as externally for academic research purposes, even though they contain personally identifiable information that could possibly compromise the privacy of patients and/or their relatives [1,2]. Personally identifiable data variables, which may be directly identifying or may offer sufficient granularity to enable re-identification of de-identified records, can include demographic data (e.g., date of birth, sex, ethnicity, language and contact information), clinical data (e.g., diagnoses, laboratory results, imaging reports and vital signs), administrative data (e.g., patient identifiers, health authority codes and marital status), next of kin information (e.g., relationship and contact details) and socio-economic data (e.g., educational background and income). Examples of re-identification through linkage of separate datasets have been provided in the work of Sweeney and colleagues, who have shown how linking public data from the Personal Genome Project, and from anonymised health records, with voter rolls and other public datasets can re-identify individuals [3,4]. A comprehensive list of personally identifiable data variables typically collected in healthcare systems is shown in Table 1.

**Table 1:** List of typical personally identifiable variables of health record data. Adapted from HL7 documentation [12] and Provincial Health Data Centre [13].

Category	Source system variables	Minimised Health Information Exchange variables
Demographic	Patient Identifier(s), National Civil Registration Number (or Passport Number), Surname, Forenames, Title, Patient Date of Birth Year, Patient Date of Birth, Ethnicity, Sex, Area of Residence, Language, Birth Name, Postal Address District of Residence, Foreign Address Indicator, Foreign Postal Address Indicator	Patient Identifiers, Surname, First Names, Date of Birth, Sex, Ethnicity, Language, Address
Clinical	Blood Group, Allergies, Medical Profile Comments, Risk Factor, Patient Death Indicator, Patient Date of Death Indicator, Patient Date of Death	Date of Birth, Date of Death, Risk Information, Blood Group
Administrative	Mobile Phone Number, Patient Street Address or Building Name, Patient Suburb, Patient Town or City, Patient Province, Patient Postcode, District of Residence Code, Health Authority Code, Patient Work Phone, Previous Surname, Patient Identification Comments, General Practitioner, Religion, Marital Status, Occupation, School, Occupation Spouse, Patient Home Phone, Patient Address Comments, Civil Registration Number Status, Patient Postal Street Address or Building Name, Patient Postal Suburb, Patient Postal Town or City, Patient Postal Province, Patient Postcode, Patient Registration Facility	Address, Contact Details
Next of kin	Next of Kin Relationship, Next of Kin Name, Next of Kin Street Address or Building Name, Next of Kin Suburb, Next of Kin Town or City Next of Kin Province, Next of Kin Postcode, Next of Kin Home Phone, Next of Kin Work Phone, Next of Kin Comments, Carer Support, Carer Name, Relationship, Relationship Internal, Carer Address, Home Phone, Work Phone, Comments	Next of Kin Details, Carer Details
Socio-economic	Place of Birth, Occupation, Education Level, Spouse Occupation, Carer Support Indicator, Area of Residence, Patient Fee Subsidy Classification	Patient Fee Subsidy Classification, Occupation, Education Level

Privacy protection is therefore essential in the preparation, management and distribution of health datasets devoid of personally identifiable information for onward use and secure data sharing within the responsible healthcare organisation, and also for repurposing of health data for use elsewhere. De-identification is a widely used privacy-preserving approach in clinical trials, for example, allowing for the removal or masking of direct identifiers while retaining coded linkages for regulatory or research follow-up [2,5]. An example of the risks of using identified data for health research was clearly demonstrated in the governance failure by University of Washington in December 2018, which led to public exposure of identified health data for 979,000 patient records being exposed online [6]. Anonymisation of large health datasets prior to research, however, irreversibly removes personal identifiers and can enable scalable and secure health data use for research by reducing privacy risks sufficiently to be granted a waiver for participants’ informed consent [1,2,7–9]. This can circumvent some of the recruitment limits, costs and potential recruitment bias associated with in-person recruitment informed consent protocols [10]. Definitions of de-identification and anonymisation are shown in Box 1. Anonymisation can add another layer to multiple data security approaches, such as governance protocols and the use of Trusted Research Environments [11], so that if one governance system fails, data protection can be upheld by the other layers of security in place. In addition, data anonymisation at source can ensure that data and platform managers do not have unnecessary views of identified data on the platforms that they manage.

**Box 1. Operational definitions of de-identification and anonymisation (from NIST SP 800-122 Guide to Protecting the Confidentiality of Personally Identifying Information [14]).**

**De-identification of Data** is used to describe records that have had enough Personally Identifying Information removed or obscured, also referred to as masked or obfuscated, such that the remaining information does not identify an individual and there is no reasonable basis to believe that the information can be used to identify an individual.

De-identified information can be re-identified (rendered distinguishable) by using a code, algorithm, or pseudonym that is assigned to individual records.

**Anonymized information** is defined as previously identifiable information that has been de-identified and for which a code or other association for re-identification no longer exists. Anonymizing information usually involves the application of statistical disclosure limitation techniques to ensure the data cannot be re-identified, such as:

- Generalising the Data—Making information less precise, such as grouping continuous values.
- Suppressing the Data—Deleting an entire record or certain parts of records.
- Introducing Noise into the Data—Adding small amounts of variation into selected data.
- Swapping the Data—Exchanging certain data fields of one record with the same data fields of another similar record (e.g., swapping the ZIP codes of two records).
- Replacing Data with the Average Value—Replacing a selected value of data with the average value for the entire group of data.

Using these techniques, the information is no longer Personally Identifying Information, but it can retain its useful and realistic properties.

There are no reported universal standards for data anonymisation or de-identification, causing some confusion and difficulty with standardising approaches [15,16]. Multiple guidelines do exist (Box 2) and key factors commonly considered include the nature and type of personal data being anonymised, as different techniques are suited to different data types and contexts; the end-user or organisation’s data analysis needs and their risk management strategies, which should include controls beyond just the anonymisation techniques; and the required utility (e.g., clarity and precision) of analysis of the anonymised data, ensuring the data remain functional for their intended purpose.

**Box 2. Examples of existing guidelines for data de-identification and anonymisation.**

- **ISO/IEC 20889:2018**—**Privacy Enhancing Data De-identification Techniques**

Description: Developed by the International Organization for Standardization (ISO), this standard defines various anonymisation and de-identification techniques.

*URL:* *https://www.iso.org/standard/69373.html*

- **GDPR Anonymisation Guidance (EU)**

Description: Under the EU General Data Protection Regulation (GDPR), anonymisation is a key mechanism for ensuring data privacy while allowing secondary use of data. The European Data Protection Board (EDPB) provides guidelines on pseudoanonymisation techniques [17].

*URL:* *https://www.edpb.europa.eu/system/files/2025-01/edpb_guidelines_202501_pseudonymisation_en.pdf*

- **NIST Special Publication 800-188**—**De-identification of Government Datasets (USA)**

Description: The National Institute of Standards and Technology (NIST) provides best practices for de-identifying datasets while preserving their usability [18].

*URL:* *https://csrc.nist.gov/pubs/sp/800/188/final*

- **The HIPAA Safe Harbor Method (USA)**

Description: The Health Insurance Portability and Accountability Act (HIPAA) in the US defines specific rules for de-identifying health data, including the Safe Harbor method, which requires removing 18 types of direct identifiers.

*URL:* *https://www.hhs.gov/hipaa/for-professionals/privacy/special-topics/de-identification/index.html*

- **OECD Guidelines on Privacy and Data Protection**

Description: The Organisation for Economic Co-operation and Development (OECD) provides high-level principles on data privacy, including anonymisation.

*URL:* *https://www.oecd.org/en/topics/privacy-and-data-protection.html*

- **UK Information Commissioner’s Office (ICO) Anonymisation Code of Practice**

Description: Provides practical guidance for organisations processing and anonymising personal data [19].

*URL:* *https://ico.org.uk/media/1061/anonymisation-code.pdf*

- **De-identification Guidelines for Structured Data, Information and Privacy Commissioner of Ontario.**

Description: Guidelines introducing the basic concepts and techniques of de-identification [20].

*URL:* *https://www.ipc.on.ca/sites/default/files/legacy/2016/08/Deidentification-Guidelines-for-Structured-Data.pdf*

The de-identification and/or anonymisation process involves a series of systematic steps, considerations and possible outcomes that are required to determine the level and types of re-identification risk involved in a dataset release, which will differ with each data release [20]. These overlapping steps from risk-based approaches can be complex and challenging, and have been compiled here through the harmonisation and mapping of multiple methodologies [20–24]. This outline of systematic steps is provided as a framework to contextualise each application of the tips provided, described in Table 2, with an indication of where our specific tips apply. We aim to provide some tips that can help to operationalise data anonymisation, whilst recognising that approaches to data anonymisation may need to be adapted to each context.

**Table 2:** Systematic and overlapping steps involved in data de-identification and anonymisation. Compiled from [20,21,23,25,26].

Step	Tips^†^	Descriptions and/or purpose	De-identification and anonymisation
Identify the relevant governance framework	1	A robust governance process for data release is described by an organisation’s data access policy and standard operating procedures, it incorporates both technical and non-technical data protection approaches and meets national legislative requirements for privacy protection.	Determine both technical and non-technical requirements that must be met in order to protect the data.
Identify risk according to the expected level of dataset exposure	1, 2	The release of either public, semi-public or non-public data determines the level of public exposure, risk and related governance or legislative requirements.	De-identification can be sufficient for non-public (internal) release, but for any semi-public or public datasets full anonymisation that removes any possibility of re-identification must be undertaken.
Determine an acceptable re-identification risk threshold	2	The re-identification risk threshold defines the minimum level of de-identification or anonymisation needed for a dataset to no longer be considered identifying personal information. It reflects an organisation’s acceptable risk level, which must usually remain very low.	The risk threshold value reflects that probability of re-identification, ranging from 0 to 1. A suitable threshold depends on expected utilisation of the data as well as context of the intended end use of the data.
Calculate the overall risk	2	The overall calculated risk represents the likelihood that one or more rows in the dataset could be re-identified in the event of a re-identification attempt. **Data risk:** The level of re-identification risk within the dataset or posed by the data release, depending on the data release model. **Context risk:** The context risk is determined by analysing potential attacks on the dataset within the chosen release model, as different models may expose the dataset to various third parties and re-identification risks.	In general: Overall risk = data risk*context risk. This requires an assessment of the data risk and the context risk; assessing the actual re-identification risk may also involve more complex calculations and probability computations.
Classify data variables (attributes)	3	In structured datasets, each row usually represents an individual, and each column corresponds to a specific variable. This step involves identifying and classifying variables (direct, indirect or quasi-identifiers) that may pose a re-identification risk and will guide subsequent processing steps.	Variables include direct, indirect or quasi-identifiers. For anonymised data, final data variables must be non-identifiers.
Data de-identification or anonymization, and assessment of privacy after processing	4, 6, 7, 8	Remove unnecessary data variables, followed by the application of appropriate techniques based on the types of identifiers and the purpose of anonymisation. Some techniques should be used in combination to enhance data protection; for example, k-anonymity is a simplified model used to measure re-identification risk and to confirm that the threshold has not been exceeded (Box 3). This step will test the efficacy of the privacy protection techniques applied. If the actual risk exceeds the threshold, stronger anonymisation or de-identification techniques must be applied until the risk is below the acceptable level [28,29].	Various techniques are used depending on the nature of the identifiers to: Remove any attribute that is clearly not required for analysis of the anonymised dataset but may increase re-identification risk, Mask direct identifiers by pseudonymization, Employ techniques such as generalisation and suppression to increase the size of equivalence classes (k-anonymisation), Ensure that the overall re-identification risk stays at or below the re-identification risk threshold, to test the effectiveness of privacy protection achieved through the applied processes.
Assess data utility after the de-identification or anonymization process	9	This process involves assessing the de-identified or anonymised dataset to confirm that its utility aligns with the intended use.	If utility is compromised, it is possible to try different techniques or combinations of techniques for privacy protection whilst improving utility—for example, adding background noise, removing re-identifying variables and blurring numerical values—which can be applied in new ways to enhance utility while maintaining the overall risk of re-identification at or below the re-identification risk threshold.
Document the process	10	Documentation is required for two important aspects: i. To manage the complexities of the sharing agreement, ensuring a common understanding among all involved parties regarding appropriate use of the data (e.g., The ADBEx Agreement builder at https://adbex-template-mou-builder.streamlit.app/) ii. To record a detailed description of data provenance and processing. Process documents must, however, be kept secure because disclosing specific parameters and algorithms could enable data re-identification through reverse-engineering by external parties.	Details of the anonymisation process, including parameters used and controls applied, should be clearly recorded for future reference. This documentation provides a consistent record of the steps, considerations and outcomes involved in de-identifying personal data and can be stored at the data source as a reference and provenance for future queries about the de-identified dataset.

†This field indicates which of the top tips provided here relate to this step.

## Ten quick tips for de-identification and anonymisation of health data

### Tip 1: Be familiar with relevant data protection legislation

Various laws outline legal frameworks for data collection, processing, use and storage and there are standards, recommendations and guidelines which vary depending on the location of data use and the applicable legal frameworks [27]. Whilst national legislature usually includes health data protection under Health Acts and Protection of Privacy Acts, some nations operate under less stringent regulations or lack comprehensive data protection laws altogether [28]. A prominent example of privacy legislation is the European Union’s General Data Protection Regulation (GDPR), which provides strong privacy protections by regulating data collection, usage and storage, while also granting individuals the right to greater control and ownership of their data, data portability and to request data deletion [29]. Under this legislation, an appropriate level of data security including the incorporation of encryption and/or pseudonymisation must be implemented to stored data from healthcare systems. As an example from the global South, the Protection of Personal Information (POPI) Act in South Africa provides for protection of personal data, including health data as ‘special personal information’, similar to the defined category of ‘special category data’ enshrined in the GDPR [30].

Understanding the limitations set by local health data protection laws can help to direct de-identification and anonymisation approaches when working with personal and health data. Identifying the individual or organisation responsible for collecting and managing the data—referred to as the ‘responsible party’ in the POPI Act and the ‘data controller’ in the GDPR, for example—can help to determine whether the data will be reused by the same responsible party within the originating secure infrastructure, which may allow for slightly less stringent requirements, or whether the data will be repurposed by another party requiring stricter anonymisation and governance.

Important considerations include whether informed consent is required to collect or access data, whether data collected for healthcare provision can be repurposed for research, and under what conditions sensitive health data may cross national borders or be stored on cloud servers outside the jurisdiction where they originated. Many countries also have additional legal protections for specific groups, such as children and minors, people with physical, intellectual, or psychosocial disabilities, prisoners, refugees and displaced persons, or populations with particular cultural or societal identities that have established rights over how their data is used—for example, the San Code of Research Ethics [31,32], or the Te Ara Tika Guidelines for Maori Research Ethics [33]. Where the application of these laws and guidelines is unclear, institutional Ethics Review Committees and legal offices may also be able to offer advice.

### Tip 2: Understand the intended data use, data-sharing model and associated level of risk

Each data release model (Table 2, step 1) allows for different modes of data sharing based on levels of data availability and protection [34], for example, publicly released and requested data may require a significant amount of de-identification to protect individual privacy due to high availability with the least amount of protection, and few restrictions on access may be required for open data. In contrast, when data are shared between institutions or specific program areas, stricter privacy and security protocols are enforced through data-sharing agreements that define usage and protections and are integral to risk mitigation strategies in non-public releases. Structured datasets refer to data in a known format and location within the data pool, such as tabular data in spreadsheets or relational databases, and other defined formats like XML, CSV, or JSON; and techniques to secure structured datasets are simpler and require no prior experience in data de-identification and anonymisation. Although privacy preservation techniques can be applied to various datasets, including static, structured, well-defined, textual and single-level datasets, we have focussed on a risk-based approach to de-identification of structured datasets, with the acceptable level of re-identification risk calculated based on the prosecutor risk factor [23].

There are always competing needs for data security and privacy compared to the need to use the data for public health purposes. A risk-based approach (Table 2, step 3), such as the Data Protection Impact Assessment (DPIA), combines de-identification or anonymisation techniques with safeguards to prevent re-identification to ensure compliance with data protection regulations, and involves calculating an acceptable level of re-identification risk for individuals before releasing a dataset. This may include calculating a *prosecutor risk factor* for situations where an external party knows a target individual is in the dataset, or a *journalist risk factor* where the inclusion of a target individual in a dataset is not known [20,21]. The prosecutor risk represents the highest risk scenario, and a conservative approach to risk calculation prioritises this score to ensure stronger privacy protection (examples in Box 3). Where data are particularly sensitive or highly granular, for example in the case of rare health conditions, true anonymisation may be difficult to achieve without losing data informativeness, so in these cases using additional layers of protection such as access control, confidentiality agreements and secure data analysis platforms may still be required to ensure protection of individuals’ anonymity.

**Box 3. Simple illustrative examples for prosecutor risk factor, journalist risk factor, context risk and overall risk calculations.**

These examples assume that direct identifiers are removed, and only quasi-identifiers remain in the dataset.

***K-anonymisation*** is a measure of the number of individual records that have exactly the same values for a defined set of variables [35].

***Equivalence classes*** are groups of records that share the same values for quasi-identifiers. Increasing k-anonymity means increasing the size of equivalence classes by de-identification and anonymisation techniques to include the required number (*k*) of individuals.

**Prosecutor risk factor (PRF):** The external party knows the individual is in the dataset of 1000 individuals and the risk depends on how unique the set of quasi-identifiers are in the dataset, i.e., the degree of k-anonymisation. The individual has the following quasi-identifiers: Age = 35 years, Sex = Female, Admission Date = 1 January 2025, Postcode = 1234. If there is one other individual in the dataset with these same quasi-identifier values, the k-anonymisation value for this set of quasi-identifiers is 2.

PRF = 1/(Total number of individuals with same quasi−identifier values)

PRF = 1/2 = 50%

Meaning the risk of re-identification of the individual is 50%.

**Journalistic Risk Factor (JRF):** The external party does not know if the individual is in the dataset and attempts re-identification by selecting records and checking for uniqueness. An analysis of the dataset shows 20 of the individuals have unique sets of quasi-identifier values (*k* = 1).

JRF = (Number of unique records)/(Total records)

JRF = 20/1000 = 2%

Meaning 2% of the records are uniquely identifiable.

**Context risk:** The probability that an attacker has the right background information or access scenario to make re-identification possible, an estimate that is based on the data sharing model. Public data has the highest risk, for example, context risk factor = 1. Controlled access data have a lower access risk value that may depend on variables such as the number of people with access, contractual and legal deterrents and the likelihood of complementary data that could permit linkage and re-identification, for example, context risk range of 0.1–0.3.

**Overall Risk Example:** Where the data risk is a prosecutor risk factor of 50%, and the context risk is calculated as 0.1 (according to El Emam’s methodology proposing a risk score of 0.1 where data have high controls and medium motive and capacity for de-identification [21]), the overall risk = 0.5 * 0.1 = 0.05.

### Tip 3: Provide a minimum dataset in line with research requirements and ethical approvals and classify identifying variables

When preparing a dataset for anonymisation and onward sharing it should, wherever possible, be tailored to a specific research question or intended use. Where applicable, the dataset should align with the ethical approval in place for the intended data use, and ethics documentation should be consulted to ensure compliance. Reviewing a provided research protocol can determine precisely which data fields are required, ensuring that only necessary variables are provided: the more variables provided per individual, the greater the risk of re-identification. In addition, if any individual has outlier values on common variables, for example, someone who is uncommonly tall, these stand-out values may make it easier to re-identify certain individuals.

Categorising variables as direct, indirect or quasi-identifiers underlies the de-identification/anonymisation process. *Direct identifiers,* such as full name, ID number and phone number, could directly and uniquely contribute to re-identification on their own, whereas *indirect identifiers,* such as age, gender, Zip code, occupation, date of birth and place of birth, are not unique on their own but could contribute to re-identification when combined with other data. *Quasi-identifiers* are indirect identifier subsets that pose a re-identification risk in combination with other (quasi-)variables, such as age + gender + zip code. A direct identifier can either be removed or replaced with a pseudonym [17], unless it holds analytical significance and is rather reclassified as a quasi-identifier and subsequently de-identified. Classifying quasi-identifiers for appropriate masking or perturbation requires predicting possible sources of background knowledge accessible to an external party, such as public registries (e.g., voter lists, court records), media (e.g., obituaries), social media, professional organisations (e.g., member lists), employers (e.g., staff directories or biographies) or individuals (e.g., neighbour, co-worker or ex-spouse), as well as data from other research projects that could also be used.

### Tip 4: Provide aggregate data where possible, ensuring sufficiently large aggregation units

For some analyses, individual data are not necessary, and aggregated datasets can be provided instead to offer a level of localised anonymity that protects against re-identification of specific individuals. For example, providing the total number of individuals requiring a particular service at a health facility may be sufficient knowledge for health service planning without needing to know the particular details of each individual. Similarly, a total count of individuals with a certain condition in a population may be sufficient for estimating prevalence without requiring individual details. The threshold of counts per the aggregation unit in use that can be shared onward or reported must be set: if a population group contains only a few people with a certain rare condition, for example, aggregated data do not provide sufficiently high counts per aggregation unit to ensure that the individuals with the condition cannot be re-identified or inferred from the dataset. Although the original dataset may not be anonymised, and may only be de-identified through the removal of directly identifying fields, the aggregated dataset to be shared onward will be truly anonymised in that it will not be possible from those data to re-identify any individual.

Aggregation by geographical region is a special case which also requires careful management and consideration, because of the risk of re-identification raised when someone’s physical location is exposed together with their sensitive data. If a geographical region such as a district, subdistrict or residential block is used as the aggregation unit for an infectious disease outbreak, for example, and there are very few cases in that region, re-identification of the individuals with the disease becomes more likely when combined with other data sources and/or local knowledge [36] and they become physically at risk because they can be found. Anecdotally, organisations usually set a minimum aggregation threshold of somewhere in the range of 15–20 counts per aggregation unit to allow onward sharing of aggregated datasets. Similarly, the individual’s data would remain potentially re-identifiable in the originating dataset, and would only be considered anonymised in the aggregated dataset, assuming sufficient minimum thresholds for aggregation.

### Tip 5: Store, compile and transfer demographic data completely separately from health data

The *data separation principle*, or a ‘data firewall’, can be applied for storage of sensitive health data with potentially identifying variants, requiring complete separation of any basic demographic or personal data that may be used to re-identify an individual, such as age, sex, education status and marital status, from sensitive data such as their clinical data. The demographic and clinical datasets are stored completely separately and can only be re-linked by the use of a *pseudonymous*, or *random identifier*, which has no meaningful relationship to the person or entity they represent. Separation of these types of data also reduces the granularity of the dataset, thus also decreasing the risk of re-identification from the exposed variables. The key linking the pseudonymous identifier to individuals is kept under high security with access by few authorised individuals. This process will not necessarily ensure the anonymity of the data, but will rather reduce the likelihood of re-identification of a de-identified dataset by data linkage.

In addition, the data separation principle may be applied not only to *data at rest*, i.e., in static storage, backup or archive files, but also *data in motion*, i.e., data being transferred from generator to end user, and *data in use*, i.e., data being used for analysis by the recipient of the anonymised dataset [37]. To this end, the separated demographic data and clinical data are stored in physically separate databases, and demographic and/or identifying data are only seen and managed by analysts who need this access in order to perform their role, for example, de-duplication of healthcare client records in a Patient Master Index. Only anonymised clinical and health-related data are accessed by researchers undertaking relevant epidemiological analyses.

Transferring datasets to end-users also requires the use of secure data transfer platforms often provided by institutions, rather than commonly-used file transfer channels such as email. Clinical and demographic files should be transferred separately from each other with distinct encryption and password protection, and passwords must be shared via a different mechanism, such as a text message, telephone call or email to a different email address; or via a secure third-party platform, for example, Bitwarden (https://bitwarden.com/).

### Tip 6: Manage precise numerical values

**Fig 1:**
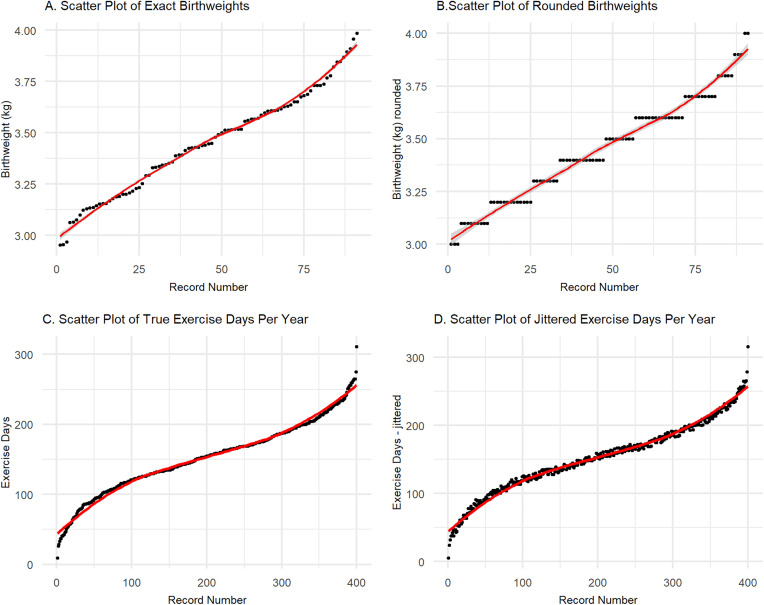
Anonymising precise numerical values. Rounding precise values to decimal places or significant figures can ensure k-anonymity is preserved whilst retaining variable characteristics and epidemiological meaning (artificial dataset). **A**: Birthweights (kg) dataset with 4-decimal place precision, **B**: Birthweights (kg) dataset rounded to one decimal place. **C**: Precise number of exercise days per year; **D**: Number of exercise days per year with jitter in range −5 to +5 days.

Avoid using highly precise data values which can be specific enough to become identifying when compared across multiple datasets. For example, a birthweight recorded to four decimal places, as noted on a child’s health card and related records, combined with a precise birthdate and facility name, could provide enough information for re-identification by cross-referencing with other data sources. Examples of precise numerical data that might present this risk include results from laboratory tests, weights, heights, age and exact count data. These data types can often be reported by rounding to a reasonable level of precision—for example, rounding birth weights to one decimal place (Fig 1A and 1B); or using clinically or biologically valid ranges and categories. Age ranges are frequently used in epidemiological analyses, and laboratory measures, for example, CD4 cell count and viral load as measures of health for people living with HIV (PLHIV), are easily binned into appropriate categories. These methods ensure that a dataset maintains a high level of epidemiological utility but remains sufficiently de-identified and preserves the required k-anonymity—which means that at least *k* records share exactly the same set of variables and cannot be distinguished from each other. By ensuring that no individual can be distinguished from the others in a group in this dataset, anonymity of each individual is achieved.

Another option for anonymising precise numerical data variables is to add ‘jitter’, or random noise to the data by adding or subtracting a sufficiently small random integer to count variables to prevent re-identification without significantly altering epidemiological or clinical inferences (Fig 1C and 1D). The size of such an integer should be context-specific, to ensure that the final number remains in the same relevant range: as an example, viral load measurements for PLHIV, an appropriate integer might fall in the range 0–250 without significantly changing the biological and clinical inferences from that metric. In some cases, the random integer may be differentially calculated to particularly protect individuals with outlier values for certain variables, taking care to ensure that where outlier values are relevant within a particular context, they retain their informativeness.

### Tip 7: Apply blurring techniques to dates

Date variables are particularly sensitive in health data, as they can be used for record linkage and re-identification by cross-referencing other datasets and clinical records. Blurring techniques can help prevent de-identification while retaining analytical value, and include generalisation, perturbation and reporting unanchored time or durations rather than specific dates.

*Generalisation* reduces the granularity of date-related data while preserving its epidemiological utility. A common approach is to report age at an event rather than exact birthdates. Age, for example, might be recorded in days for neonates (up to 2 weeks), weeks for newborns (up to 8 weeks), months for infants (up to 24 months) and in years thereafter, to ensure that early-life health trends can be analysed without disclosing precise birthdates.

*Perturbation* involves introducing a small, undisclosed modification to date variables, such as shifting all dates within a dataset by a fixed but unknown number of days. The shift should be small enough to retain epidemiological and seasonal patterns but large enough to prevent re-identification through record linkage. This method is particularly useful in longitudinal health studies where exact timing is less critical than overall trends. In some use cases where epidemiological inferences can still be retained, a random integer can be assigned per individual rather than to the entire dataset, to further reduce the possibility of reverse-engineering the de-identification process.

Providing less specific date information is another effective strategy: instead of recording full birthdates, for example, datasets need only include only the year of birth. Similarly, age in years at death, year of death, or time to death after an event may be reported rather than the exact date of death, ensuring that mortality data remain useful for health research while reducing the risk of individual re-identification.

### Tip 8: Engage a trusted third party for linkage and anonymisation of sensitive identified datasets

Linkage of datasets is a special case which may sometimes be needed, and where protection of privacy can be difficult due to restrictions on sharing of identified data. When combining two datasets that require the use of direct identifiers to ensure accurate linkage but need to be provided in an anonymised format, a **trusted third party** can provide an effective and secure solution to generate the linked, anonymised dataset [38]. They are not affiliated with the responsible party for either dataset, have no vested interest in the resulting dataset and act as a neutral intermediary. The trusted third party receives two identified datasets, links them using identifying variables and then proceeds with anonymisation and perturbation steps to ensure compliance with data protection regulations.

To maintain the highest standards of confidentiality and security, the trusted third party must sign a formal **non-disclosure agreement**, committing to the deletion of all datasets, both identified and anonymised, once the process is completed. Data Linkage Services in Western Australia is an example of a mature trusted third party that links and anonymises health data from various sources to support research and policy development [39,40].

### Tip 9: Test the dataset utility and k-anonymisation

**Fig 2:**
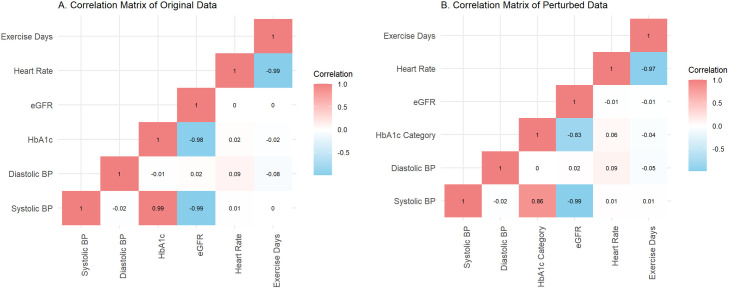
Checking bivariate correlation before and after perturbation. An exhaustive bi-variate correlation matrix shows that the bivariate correlation relationships remain generally similar despite perturbation. Red shading indicates positive correlation, blue shading indicates negative correlation. Values within each cell show the correlation coefficient. **A**: Original dataset, **B**: Dataset after perturbation of multiple fields.

To ensure data utility while maintaining privacy, it is important to evaluate the effects of anonymisation and perturbation on the structure of the dataset. This can be achieved by conducting simple descriptive analyses of the dataset before and after the anonymisation process, as well as checking multivariate relationships and data structure. Here, we show a simple example of checking variable distributions before and after these processes (Fig 1) and generating an exhaustive bivariate correlation matrix for numerical variables to compare pre- and post-anonymisation metrics (Fig 2).

**Fig 3:**
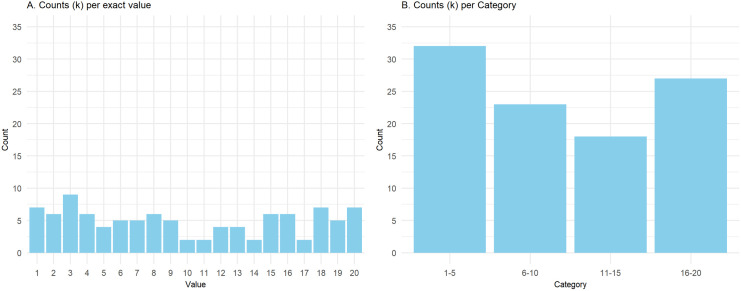
Checking k-anonymisation before and after perturbation. Creating categories based on numerical value range can increase k-anonymity (artificial dataset with x-axis = value/category, and y-axis = counts per value/category). **A**: Exact integer variables ranging from 1 to 20, **B**: Categorical variables derived from integer variables ranging from 1 to 20.

It is also possible to assess k-anonymisation by counting the number of records per potentially identifying variable, for example, showing the increase in k-anonymisation by binning numerical variables into categories defined by range (Fig 3).

These checks also make it possible to check the balance between data utility and risk of exposure, for example, by checking how generalised the data has become using metrics like k-anonymity and comparing these to data outputs such as descriptive comparisons, relationship preservation and predictive accuracy, before and after perturbation. Where data utility has been too compromised through anonymising approaches to be useful for the intended purpose, it is advisable to consider different anonymisation techniques that may better preserve the informativeness of the data. Whilst we have focussed here on k-anonymity and related metrics, additional methods may also be considered for assessing anonymity, each bringing their own strengths and challenges. Examples include metrics such as l-diversity, t-closeness and differential privacy, reviewed in [41] with extensive examples of implementation also included. There are also an increasing number of open-source tools that can be used to evaluate privacy risk, such as ARX [42] or the sdcMicro Package [43].

### Tip 10: Before data transfer, sign a data sharing agreement or legal document detailing the onward use of the data

A signed Memorandum of Understanding, Data Access Agreement or Data Transfer Agreement, or a formal legal agreement that outlines the terms of the data sharing arrangement can formalise the terms of sharing and avoid misunderstandings between parties. This agreement should detail how the dataset will be securely transmitted between parties, and how the data will be used, ensuring this aligns with permissions and ethical approvals.

The data sharing agreement should also describe any restrictions on its further dissemination and the terms and timeline for data deletion. Any intellectual property considerations should be clearly defined, addressing ownership rights and any applicable restrictions, with an outline for how to attribute the data originators in publications and research outputs. A plan for conflict resolution should also be included and specify which jurisdiction will apply in overseeing the dataset, particularly when data cross borders. Finally, it is important to clarify whether any cost recovery or benefit sharing is required as part of the agreement, ensuring that all parties are aware of financial or other obligations. An example of an online app to create a fit-for-purpose data sharing agreement (https://adbex-template-mou-builder.streamlit.app/, Tamuhla and colleagues, 2025, *Manuscript in Preparation;* and [34]) incorporates user data to generate a draft data sharing document containing appropriate clauses which can be refined further and/or shared with an institutional Technology Transfer Office or Legal Department to draw up a legal agreement where required. Existing international data stewardship guidelines, such as FAIR [44], CARE [45] and GA4GH [46] frameworks also provide a toolkit to inform and facilitate best practices in data sharing.

In compiling these practical tips, one important area in which we have noted a significant absence of guidance, tools and methodologies in common use for data protection is in the area of dataset deletion, where data use is granted for a certain period of time and then—according to the signed data sharing agreement—must be deleted. Whilst some digital rights management commercial software may build in time-sensitive data and document locks on documents and occasionally datasets, these are not in common use for data governance of health data. This is an area that we would recommend requires significant work to operationalise time limit enforcement on datasets for re-use, and to augment dataset security for onward sharing.

## Conclusion

Data de-identification and anonymisation are essential for protecting patient privacy in healthcare systems and facilitating data sharing in health research. While there are no universal standards and the processes can be technically complex, here we provide a summarised framework describing the steps in this process and offer ten practical tips to help to guide effective application data de-identification and anonymisation whilst following these steps. The tips we provide are intended to provide practical, operational guidance to simplify the process of protecting privacy of individuals through de-identification and anonymisation of health data, whilst recognising the need to maintain data utility and relevant information.
